# Ambulatory Blood Pressure Monitoring as a Useful Tool in the Cardiological Assessment of Pancreas Transplant Recipients with Type 1 Diabetes

**DOI:** 10.3390/diagnostics13172724

**Published:** 2023-08-22

**Authors:** Małgorzata Buksińska-Lisik, Przemysław Jerzy Kwasiborski, Robert Ryczek, Wojciech Lisik, Artur Mamcarz

**Affiliations:** 1 3rd Department of Internal Medicine and Cardiology, Medical University of Warsaw, 2 Bursztynowa St., 04-749 Warsaw, Poland; 2 Department of Cardiology and Internal Medicine, Multidisciplinary Hospital Warsaw Miedzylesie, 2 Bursztynowa St., 04-749 Warsaw, Poland; 3 Department of Cardiology and Internal Diseases, Military Institute of Medicine, 128 Szaserów St., 04-141 Warsaw, Poland; 4 Department of General and Transplantation Surgery, The Medical University of Warsaw, 59 Nowogrodzka St., 02-006 Warsaw, Poland

**Keywords:** type 1 diabetes, hypertension, ABPM, pancreas transplantation, pancreas–kidney transplantation, coronary artery disease

## Abstract

Having the appropriate tools to identify pancreas recipients most susceptible to coronary artery disease (CAD) is crucial for pretransplant cardiological assessment. The aim of this study is to evaluate the association between blood pressure (BP) indices provided by ambulatory blood pressure monitoring (ABPM) and the prevalence of CAD in pancreas transplant candidates with type 1 diabetes (T1D). This prospective cross-sectional study included adult T1D patients referred for pretransplant cardiological assessment in our center. The study population included 86 participants with a median age of 40 (35–46) years. In multivariate logistic regression analyses, after adjusting for potential confounding factors, higher 24 h BP (systolic BP/diastolic BP/pulse pressure) (OR  =  1.063, 95% CI 1.023–1.105, *p* = 0.002/OR = 1.075, 95% CI 1.003–1.153, *p* = 0.042/OR = 1.091, 95 CI 1.037–1.147, *p* = 0.001, respectively) and higher daytime BP (systolic BP/diastolic BP/pulse pressure) (OR  =  1.069, 95% CI 1.027–1.113, *p* = 0.001/OR = 1.077, 95% CI 1.002–1.157, *p* = 0.043/OR = 1.11, 95% CI 1.051–1.172, *p* = 0.0002, respectively) were independently and significantly associated with the prevalence of CAD. Daytime pulse pressure was the strongest indicator of the prevalence of CAD among all analyzed ABPM parameters. ABPM can be used as a valuable tool to identify pancreas recipients who are most susceptible to CAD. We suggest the inclusion of ABPM in pretransplant cardiac screening in type 1 diabetes patients eligible for pancreas transplantation.

## 1. Introduction

Pancreas transplantation is a well-established treatment option for selected patients with diabetes type 1 (T1D) that results in restoring glycemic control, improves the quality of life, and ameliorates diabetic complications [1]. Diabetic patients are qualified for different types of transplantation based on their kidney function. Patients with preserved renal function receive pancreas transplant alone (PTA), and patients with end-stage diabetic kidney disease receive SPK (simultaneous pancreas–kidney transplantation) or cadaver/life kidney transplantation (KTA) and then pancreas after kidney transplantation (PAK) [2].

Regardless of the type of surgery, the success of transplantation is at first determined by the perioperative course. In-hospital cardiovascular complications are an important problem among non-surgical complications associated with pancreas transplantation [3,4]. The main reason is the high prevalence of atherosclerotic disease in overall pancreas recipients. Coronary artery disease and/or peripheral vascular disease are reported in pretransplant assessment of recipients in 47% of SPK and 24% of PTA [5]. The crucial causes of the accelerated progression of atherosclerosis in diabetic patients are hyperglycemia and glycemic variability [6]. However, the prevalence of classical cardiovascular risk factors in overall T1D patients and pancreas–kidney recipients is very high, and they play an important role in the progression of coronary artery disease (CAD) [7,8,9]. Hypertension is observed in the majority of pancreas transplant candidates (both SPK and PTA) and presents likely the most common cardiovascular risk factor in this group of patients [4,10]. However, which blood pressure parameter is most closely associated with CAD in pancreas transplant candidates remains unclear.

Ambulatory blood pressure monitoring (ABPM) provides a multitude of measurements and seems to be an appropriate tool to solve this problem. The use of ABPM to confirm the diagnosis of hypertension and quantifying the effects of treatment is supported by European and American Guidelines [11,12]. Moreover, ABPM provides the additional index of prognostic value, making it an effective predictor of cardiovascular outcomes in various populations [13,14,15,16].

The relatively long waiting time for pancreas transplantation provides ample opportunity for intensive screening and adequate interventions to optimize blood pressure control, thus reducing the risk of CAD and subsequent risk of perioperative coronary events. Therefore, it is very important to choose the best CAD indicators from the wide range of measurements provided by ABPM.

Therefore, this study aimed to evaluate the association of blood pressure (BP) indices with the prevalence of CAD in pancreas transplant candidates.

## 2. Materials and Methods

### 2.1. Study Design and Population

This prospective cross-sectional study was conducted at the 3rd Department of Internal Medicine and Cardiology, Medical University of Warsaw. The study population included adult T1D patients eligible for pancreas transplantation (PTA/SPK), who were referred for pretransplant cardiological assessment in our center from August 2018 to August 2022. The exclusion criteria included the following: type 2 diabetes; sleep apnea; severe valvular heart disease; heart failure; coronary artery disease; documented arrhythmia; and changes in lipid-lowering and/or in antihypertensive therapy within 3 months before the study entry. The study protocol was conducted following the Declaration of Helsinki and was approved by the local Bioethics Committee at the Medical University of Warsaw, Poland (no. KB/115/2018). All participants signed an informed consent form to participate in the study.

### 2.2. Measurements and Definitions of Variables

The following demographic and clinical variables were collected: age; sex; diabetes duration; renal replacement therapy; type of planned transplantation procedure (PTA/SPK); smoking status; hypertension; dyslipidemia; and the use of antihypertensive and lipid-lowering drugs. Hypertension was defined as systolic blood pressure (SBP) > 140 mmHg or diastolic blood pressure (DBP) > 90 mmHg and/or a patient on antihypertensive therapy before admission. Dyslipidemia was diagnosed if at least one of the following values was met: total cholesterol (TC) ≥ 5.0 mmol/L; low-density lipoprotein-cholesterol (LDL-C) ≥ 3.0 mmol/L; triglycerides (TG) ≥ 1.7 mmol/L; and if a patient was on lipid-lowering therapy. Current smoking was defined as active cigarette smoking in the last 5 years before inclusion in the study.

All participants were weighed after overnight fasting and dialysis patients were weighed on their non-dialysis day. Body mass index (BMI) was calculated as weight [kg] divided by the square of height [m]. Obesity was defined as a BMI ≥ 30 kg/m^2^.

Blood samples were collected after overnight fasting. The concentrations of glycated hemoglobin (HbA1c), creatinine, TC, high-density lipoprotein-cholesterol (HDL-C), and TG were measured using a commercially available analyzer (Beckman Coulter, Inc., Brea, CA, USA). The concentration of LDL-C was calculated according to the Friedewald formula [17].

AMBP was recorded in all participants using the validated oscillometric device (90217, Spacelabs Healthcare, Inc., Snoqualmie, WA, USA) in agreement with the current guidelines of the European Society of Hypertension (ESH) [18]. Measurements started between 10 and 11 AM and lasted 24 h. Appropriated-sized cuffs were placed on the nondominant arm. In dialysis patients, the test was performed on a non-dialysis day on an arm without a dialysis fistula. During the day, measurements were scheduled every 20 min, and at night (10.00 p.m.–6.00 a.m.), they were scheduled every 30 min. If the number of useless blood pressure (BP) records exceeded 30%, the measurement session was excluded and the patient was consequently excluded from statistical analyses. Median systolic blood pressure (SBP), diastolic blood pressure (DBP), and pulse pressure (PP) were calculated separately for daytime, night-time and over 24 h. PP was calculated as SBP—DBP. Blood pressure load was calculated separately for systolic and diastolic BP as the percentage of elevated pressures above a defined threshold value. The 24 h systolic blood pressure load (SBPL) was defined as the percentage of systolic readings more than 130 mmHg, and the 24 h diastolic blood pressure load (DBPL) was defined as the percentage of diastolic blood pressures of more than 80 mmHg. Nocturnal BP dipping status was defined based on the dipping ratio calculated as mean night-time SBP divided by mean daytime SBP. Depending on this value, participants were defined as dippers (dipping ratio ≤ 0.9), non-dippers (dipping ratio > 0.90 to ≤1.00), and reverse dippers (dipping ratio > 1) [13]. A mean 24 h value of <130/80 mmHg was used as the therapeutic target based on extrapolation from office BP measurements as ABPM targets to be achieved by treatment have not yet been determined [19].

### 2.3. Assessment of CAD

CAD was evaluated based on stress tests and invasive coronary angiography. Patients with a high clinical likelihood of CAD—with long-standing diabetes (T1D duration ≥ 20 years) and/or severe chronic kidney disease (eGFR < 30 mL/min/1.73 m^2^)—were directly referred for coronary angiography. The other patients underwent stress tests, which included an exercise stress test on a treadmill (EST) or pharmacologic stress test. EST was performed in patients with a normal resting electrocardiogram who were able to exercise adequately. The patients exercised according to the standard Bruce protocol, which consists of 3 min stages with increased speed and incline of the treadmill at each stage. Patients with physical limitations or inconclusive results of EST were subjected to a pharmacologic stress test using dipyridamole 99 mTc-sestamibi single-photon emission computed tomography (SPECT). Positive results on stress tests uniformly led to invasive coronary angiography. Coronary angiography was used instead of coronary CT angiography due to patients’ risk profile, high likelihood of extensive coronary calcification (due to CKD), local expertise, and availability. Coronary angiography was performed using a Philips Allura Xper DF20 X-ray system with standard diagnostic catheters through radial access. The obstructive CAD was defined as at least one ≥50% lesion in an artery with ≥2 mm caliber.

### 2.4. Statical Analysis

Categorical variables were presented as number and percentage of distribution and continuous data as median with interquartile range (IQR). The Shapiro–Wilk test was used to test the normality of the data distribution. For parameters without normal distributions, statistical analyses were based on non-parametric tests. The Mann–Whitney U test was used to compare numerical variables between two groups, and the Fisher’s exact test or chi-squared test was used to examine the relationship between categorical variables. The correlation between ABPM parameters and CAD and between ABPM parameters and potential predictors of CAD (included in the regression analysis) was checked using Spearman’s correlation coefficient (r). The following classification of the correlation strength was used: 0.0 ≤ |r| ≤ 0.2, no correlation; 0.2 ≤ |r| ≤ 0.4, low correlation; 0.4 ≤ |r| ≤ 0.7, moderate correlation; 0.7 ≤ |r| ≤ 0.9, high correlation; 0.9 ≤ |r| ≤ 1.0, very high correlation.

A multivariate logistic regression analysis was used to test the combined relationship between the presence/absence of CAD and selected ABPM parameters adjusted for confounding factors, including age, hypertension, smoking, TG, statins use, and hemodialysis. Each model consists of only one of the parameters from ABPM (e.g., 24 h SBP or 24 h DBP, etc.) and the same set of potential confounding factors. The multivariate model used the backward, stepwise elimination method, starting with a model including all the variables. The results were presented as odds ratio (OR) with a 95% confidence interval (CI). Statistical analyses were performed using Statistica version 13.3 (TIBCO Software Inc., Palo Alto, CA, USA). For all statistical analyses, a *p*-value < 0.05 was considered significant.

## 3. Results

### 3.1. Participant Characteristics

The study population included 89 patients, but 3 of them were excluded from further analysis because the number of useless ABPM readings exceeded 70%. The statistically analyzed study population included 86 participants with a median age of 40 (35–46) years, 41 (47.7%) of whom were men. The baseline characteristics of the participants are illustrated in Table 1.

The median duration of T1D was 26 (22–31) years, and most of the participants (*n* = 70; 81.4%) had long-standing diabetes (duration > 20 years). Depending on kidney function, patients qualified for PTA (*n* = 25; 29.1%) or SPK (*n* = 61; 70.9%). The majority of SPK candidates were on HD (*n* = 52; 85.3% out of SPK), whereas nine patients (14.7% out of SPK) were qualified for preemptive transplantation. The prevalence of classical cardiovascular risk factors was very high, and hypertension was the most common CRF in our cohort (*n* = 70; 81.4%). The majority of hypertensive patients reported the use of antihypertensive drugs (*n* = 54; 77.1% of hypertensive patients) and the majority of dyslipidemic patients reported the use of statins (*n* = 37; 82.2%).

### 3.2. CAD and Cardiovascular Risk Factors

Most participants (*n* = 81; 94.1%) were referred directly for coronary angiography, 86.4% of them (*n* = 70) due to long-standing diabetes (T1D duration ≥ 20 years) and 13.6% of them (*n* = 11) due to severe chronic kidney disease (eGFR < 30 mL/min/1.73 m^2^). The other patients (*n* = 5) underwent stress tests: two patients had EST, two patients had SPECT, and one patient had both EST and SPECT. Out of all the stress tests, three were reported as negative and two were reported as positive. Positive stress tests led to invasive coronary angiography.

Finally, coronary angiography was performed in 83 patients (96.5% of the entire cohort). CAD was diagnosed in 30.2% of participants (*n* = 26). According to the Heart Team decision, one person (3.8% out of patients with CAD) was disqualified from pancreas transplantation due to diffuse coronary lesions unsuitable for any method of revascularization; four patients (15.4% out of patients with CAD) qualified for CABG; and 8 (30.8% out of patients with CAD) underwent PCI. Half of the patients with CAD (*n* = 13) qualified for optimal medical therapy and were approved for pancreas transplantation. Patients subjected to revascularization will be reevaluated as candidates for pancreas transplantation after invasive treatment of CAD.

The differences in cardiovascular risk factors between patients with and without CAD are shown in Table 1. Hypertension, dyslipidemia, and hemodialysis were significantly more common in patients with CAD than in patients without. Patients with CAD had a significantly higher concentration of TG and a lower concentration of HDL-C than those without CAD, but there were no significant differences between the levels of TC and LDL-C. There were no significant differences in sex, BMI, smoking, duration of T1D, and level of HbA1C in the analyzed subgroups.

### 3.3. CAD and ABPM Components

**Figure 1:**
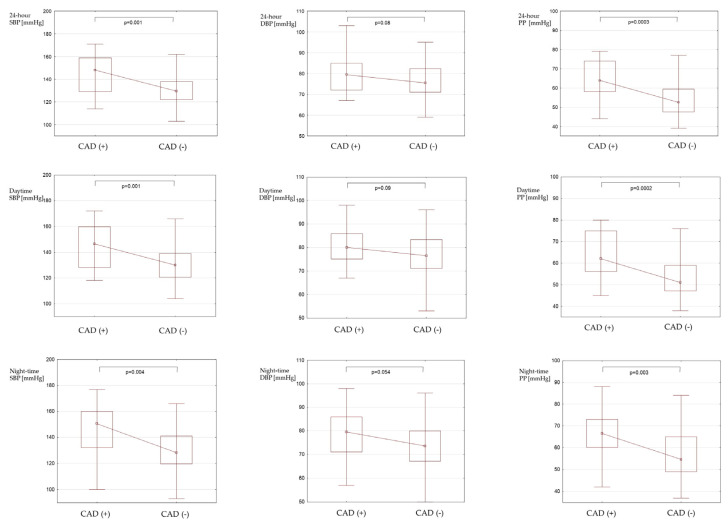
Blood pressure components in patients with CAD and without CAD. Continuous variables are presented as median with interquartile range (IQR). CAD: coronary artery disease; SBP: systolic blood pressure; DBP: diastolic blood pressure; PP: pulse pressure (SBP—DBP).

The median 24 h BP was 131.5 mmHg (124–147) and exceeds the therapeutic goal (24 h BP < 130/80 mmHg) in 53.5% of the entire cohort (*n* = 46) and as much as in 73% of patients with CAD (*n* = 19). The 24 h SBPL was significantly higher in patients with CAD than in patients without CAD (53.5% (31–91) vs. 33% (12–51), *p* = 0.002). There were no significant differences in 24 h DBPL between the analyzed groups (Supplementary Table S1). Patients with CAD had significantly higher SBP (24 h, daytime, and night-time) and higher PP (24 h, daytime, and night-time) than patients without CAD. There were no differences in DBP between patients with and without CAD. BP components in patients with and without CAD are presented in Figure 1.

There were no significant differences between nocturnal dipping status (dippers/non-dippers/revers dippers) and the prevalence of CAD in the study group (Table 2).

Spearman’s correlation analysis was used for testing the correlation between selected ABPM parameters and CAD. Based on Spearman’s correlation matrices, we found a significant, weak correlation between both 24 h SBP and 24 h SBPL and CAD. There was no significant correlation between 24 h DBP and between 24 h DBPL (Supplementary Table S2). In addition, Spearman’s correlation analysis was used to test the correlation between ABPM parameters and factors included as CAD predictors in the regression analysis. We found a significant weak to moderate correlation between hemodialysis and all 24 h BP values (SBP, DBP, PP), except daytime DBP. Moreover, we found a significant weak correlation between smoking and both 24 h SBP and DBP and daytime SBP. There was a weak significant inverse correlation between age and 24 h and daytime DBP and weak positive correlation between age and all PP values (Supplementary Table S3).

In multivariate logistic regression analyses, after adjusting for potential confounding factors (age, smoking, hypertension, statins using, TG, hemodialysis), higher 24 h BP (SBP, DBP and PP) (OR  =  1.063, 95% CI 1.023–1.105, *p* = 0.002 and OR = 1.075, 95% CI 1.003–1.153, *p* = 0.042 and OR = 1.091, 95 CI 1.037–1.147, *p*= 0.001, respectively), and higher daytime BP (SBP, DBP and PP)(OR  =  1.069, 95% CI 1.027–1.113, *p* = 0.001 and OR = 1.077, 95% CI 1.002–1.157, *p* = 0.043, and OR = 1.11, 95% CI 1.051–1.172, *p* = 0.0001, respectively) were significantly and independently associated with the prevalence of CAD. Daytime PP was the strongest indicator of the prevalence of CAD among all analyzed ABPM parameters. When daytime PP increased by 1 mmHg, the odds of having CAD increased 1.11 times. No significant association was found between night-time BP (SBP, DBP, PP) and the prevalence of CAD (Table 3).

## 4. Discussion

In the present study, we found hypertension to be the most common traditional risk factor among potential pancreas recipients with T1D. The main finding is a strong association between specific BP indices provided by ABPM and the prevalence of CAD. In T1D patients referred for pancreas transplantation, the higher 24 h and higher daytime median BP values (SBP, DBP, PP) were significantly associated with the higher prevalence of CAD. The associations remained significant after adjusting for well-known cardiovascular risk factors, including hemodialysis. Daytime PP was the strongest indicator of the prevalence of CAD in our cohort. No significant association was found between night-time BP (SBP, DBP, PP) and the prevalence of CAD. Moreover, there was no significant correlation between CAD and BP dipping status. In addition, we identified that BP values exceeded the therapeutic goal in the majority of the enrolled patients, even though they were on antihypertensive therapy.

CAD was found in one third of the study population. The prevalence of CAD in our population was relatively low, although the study group was exposed to a very high cardiovascular risk. Our finding is opposite to that of St Michel D. et al., who detected discrete evidence of CAD in 71.7% of T1D candidates for SPKT subjected to coronary angiography. However, when considering all their T1D patients qualified for SPKT, CAD was diagnosed in 47.5% (28 out of 59 subjects) [20]. Similarly, Marques J. et al. reported CAD in 48.1% of SPKT recipients subjected to coronary angiography, but in fact, CAD was diagnosed in 37.4% of the entire cohort (37 out of 99 subjects) [21]. These differences may be caused by several reasons. The first reason is the difference in the analyzed populations. In our study, 70% of participants qualified for SPK and 30% qualified for PTA, whereas the mentioned authors included only SPK recipients. The second reason is that the adopted definition of CAD focused only on obstructive coronary artery disease. 

In our study, there was a similar prevalence of CAD in women and men. Our results are in line with other researchers who have found that diabetic women lose their female protection against cardiovascular disease [22,23,24]. Our analysis has shown that the prevalence of traditional cardiovascular risk factors (hypertension, dyslipidemia, smoking) was very high. All these factors are partially related to the progression of atherosclerosis in patients with T1D [25]. Moreover, our results proved that hemodialysis was closely associated with the prevalence of CAD in this population. Our findings are in line with Tonelli M et al. who have linked uremia-specific factors with CAD in chronic kidney disease and hemodialysis [26]. Excessive risk of cardiovascular events in our patients results from both long-term diabetes and chronic kidney disease, which magnifies the risk [27,28,29].

In addition, we demonstrated that in more than half of the entire cohort, BP values exceeded the therapeutic goal, even though the patients were undergoing antihypertensive therapy. The number of people with insufficient BP control among patients with CAD was much higher and exceeded 70%. These results demonstrate that most patients were treated unsuccessfully, likely due to the lack of a valuable monitoring tool.

Our analysis has shown that higher 24 h and daytime median BP values (SBP, DBP, PP) were significantly associated with the higher prevalence of CAD. Moreover, we confirmed that each of the above BP parameters was an independent predictor of the increased probability of the prevalence of CAD after adjusting for potential confounding factors. Our results demonstrate that strict control of BP values is crucial in this population. The most impressive result was that daytime PP was the strongest independent predictor of the prevalence of CAD in our cohort. PP is a surrogate indicator of arterial stiffness [30,31]. Arterial stiffness is a natural degenerative process associated with aging. Additionally, this process is accelerated by various conditions, including diabetes and chronic kidney disease, resulting in increased the risk of coronary events in these patients [32]. According to Rönnback M et al., patients with T1D have a higher PP that increases in earlier age compared with the nondiabetic population, and PP increases at an earlier age than in the general population. The authors suggest that the higher cardiovascular risk of T1D patients is a result of accelerated arterial stiffening [33]. Other researchers demonstrated that arterial stiffness is associated with vascular complications and predicts mortality in T1D [34,35]. PP as an indirect exponent of arterial stiffness is a noteworthy parameter, regardless of whether it is a predictive factor or only a marker of CAD in this group of patients.

We did not find any significant associations between night-time BP and the prevalence of CAD. Furthermore, there were no significant differences between nocturnal dipping status and the prevalence of CAD, either. Our findings are in contrast to some studies showing that the non-dipper pattern is associated with a higher cardiovascular risk [36,37,38]. However, some researchers have proven that classification according to the dipping status is only slightly repetitive [39,40]. Moreover, Burgos-Alonso N at al. demonstrated that among diabetics, the diagnostic reproducibility is even lower [41]. We suggest that the use of antihypertensive drugs and the low reproducibility of nocturnal dipping patterns in diabetic patients can likely explain our findings.

Pancreas transplantation is a high-risk surgery because of the risk of adverse coronary events. Vigorous cardiological pretransplant assessment is a key to the identification of those particularly vulnerable to cardiovascular complications. The average waiting time for a pancreas transplantation (PTA/SPK) is one to two years [42]. Thus, the long waiting time allows abnormalities to be detected and appropriate treatment to be applied. We implement very complex and cost-consuming procedures for cardiological patients’ assessment for organ transplantation. However, due to the long waiting time, all tests must be repeated several times. Therefore, it is worth considering a relatively inexpensive, non-invasive, outpatient diagnostic method that can simplify pretransplant cardiological assessment. Given that hypertension is a fully modifiable risk factor, it seems reasonable to use data from ABPM to initiate and monitor antihypertensive treatment. ABPM detects patients who are both under-treated for hypertension and who are most susceptible to CAD among potential pancreas recipients. We suggest that ABPM can be used as a valuable screening tool to assess and modify the risk of CAD and likely the risk of perioperative coronary events in pancreas recipients. Further studies on the relationship between ABPM values and perioperative coronary events are necessary to establish the relevance of ABPM in pretransplant cardiological assessment.

Our study has some limitations; first is the small sample size. However, the study group is fully representative because of the low number of patients referred for pancreas transplantation in our country. The second limitation of our study is its cross-sectional nature, which limits the ability to establish causal relationships. Third, the definition of daytime and night-time were determined according to clock time, regardless of the actual activity of the patient. However, Dimsdale et al. demonstrated adequate reliability between the BP results regardless of the method used to determine night-time [43]. Another limitation of the present study is the use of statins and antihypertensive drugs, which may have confounded the presented findings. However, our study aimed to evaluate the associations of BP indices with the prevalence of CAD in the precisely determined cohort, so we were interested in the real population without any selection bias.

## 5. Conclusions

Hypertension is a very important cardiovascular risk factor in candidates for pancreas transplantation. ABPM can be used as a valuable screening tool to identify patients who are most susceptible to CAD. We suggest the inclusion of ABPM in pretransplant cardiac screening in T1D patients eligible for pancreas transplantation.

## Figures and Tables

**Table 1:** Characteristics of the study population stratified by presence/absence of CAD.

	Total (*n* = 86)	CAD (*n* = 26)	No CAD (*n* = 60)	*p*-Value
Age [years]	40 (35–46)	43.5 (38–51)	39 (34–45)	**0.03**
Sex [Male]	41 (47.7%)	15 (57.7%)	26 (43.3%)	0.2
SPK	61 (70.9%)	23 (88.5%)	38 (63.3%)	0.02
PTA	25 (29.1%)	3 (11.5%)	22 (36.7%)	
Duration of T1D [years]	26 (22–31)	27 (24–34)	25 (22–29)	0.08
Hemodialysis	52 (60.5%)	22 (84.6%)	30 (50%)	**0.004**
Hypertension	70 (81.4%)	25 (96.1%)	45 (75%)	**0.03**
ACEi/ARBs	41 (47.7%)	20 (77%)	21 (35%)	**0.0004**
Calcium channel blockers	52 (60.5%)	19 (73%)	33 (55%)	0.15
Beta-blockers	46 (53.5%)	19 (73%)	27 (45%)	**0.02**
Diuretics	47 (54.7%)	16 (61.5%)	31 (51.7%)	0.5
Alpha-blockers	13 (15.1%)	5 (19.2%)	8 (13.3%)	0.5
Centrally acting agents	6 (7%)	2 (7.7%)	4 (6.7%)	0.9
Current smoking	25 (29%)	11 (42.3%)	14 (23.3%)	0.1
Dyslipidemia	45 (52.3%)	19 (73.1%)	26 (43.3%)	**0.02**
Statins using	37 (43%)	15 (57.7%)	22 (36.7%)	0.1
BMI [kg/m^2^]	23.1 (20.8–25.8)	23.8 (20.6–26.5)	22.9 (20.8–25.4)	0.6
HbA1c [%]	7.45 (6.8–8.22)	7.71 (7.2–8.1)	7.37 (6.74–8.28)	0.2
TC [mmol/L]	4.75 (3.8–5.7)	5.1 (3.6–6)	4.7 (3.9–5.7)	0.8
LDL-C [mmol/L]	2.55 (2–3.2)	2.7 (1.8–3.5)	2,5 (2.1–3.1)	0.7
HDL-C [mmol/L]	1.4 (1.2–1.9)	1.3 (1.2–1.4)	1.5 (1.2–1.95)	**0.036**
TG [mmol/L]	1.35 (1–1.8)	1.8 (1.3–2.1)	1.2 (0.9–1.7)	**0.0007**

Categorical variables are presented as numbers and percentages (%), and continuous variables are presented as median with interquartile range (IQR). CAD, coronary artery disease; SPK, simultaneous pancreas–kidney transplantation; PTA, pancreas transplant alone; T1D, type 1 diabetes; ACEi, angiotensin-converting enzyme inhibitors; ARB, angiotensin receptor blockers; BMI, body mass index; HbA1c, glycated hemoglobin; TC, total cholesterol; LDL-C, low-density lipoprotein cholesterol; HDL-C, high-density lipoprotein cholesterol; TG, triglycerides. Significant differences are marked in bold.

**Table 2:** Dipping status in patients with CAD and without CAD.

	Total (*n* = 86)	CAD (*n* = 26)	No CAD (*n* = 60)	*p*-Value
Dippers	13 (15.1%)	6 (23.1%)	7 (11.7%)	0.4
Non-dippers	36 (41.9%)	9 (34.6%)	27 (45%)	
Revers dippers	37(43%)	11 (42.3%)	26 (43.3%)	

Categorical variables are presented as numbers and percentages. CAD: coronary artery disease; dippers: participants with normal SBP dipping status (a ratio of night-to-daytime SBP ≤ 0.9); non-dippers: participants with non-dipping SBP status (a ratio of night-to-daytime SBP > 0.90 to ≤1.00); revers dippers: participants with reverse dipping SBP status (a ratio of night-to-daytime SBP > 1).

**Table 3:** Association between ABPM parameters and the prevalence of CAD.

Variables		Crude OR (95% CI)	*p*-Value	Adjusted OR (95% CI)	*p*-Value
SBP [mmHg]	24 h	1.054 (1.022–1.088)	**0.001**	1.063 (1.023–1.105)	**0.002**
	Daytime	1.056 (1.024–1.09)	**0.0006**	1.069 (1.027–1.113)	**0.001**
	Night-time	1.035 (1.01–1.061)	**0.0065**	1.024 (0.994–1.055)	0.1
DBP [mmHg]	24 h	1.059 (1.003–1.117)	0.0578	1.075 (1.003–1.153)	**0.042**
	Daytime	1.053 (0.999–1.11)	0.0541	1.077 (1.002–1.157)	**0.043**
	Night-time	1.043 (0.999–1.088)	0.0538	1.038 (0.982–1.097)	0.18
PP [mmHg]	24 h	1.078 (1.029–1.128)	**0.0014**	1.091 (1.037–1.147)	**0.001**
	Daytime	1.092 (1.042–1.145)	**0.0003**	1.11 (1.051–1.172)	**0.0002**
	Night-time	1.056 (1.015–1.1)	**0.0073**	1.033 (0.986–1.83)	0.17

Multivariate logistic regression analysis with backward, stepwise elimination was used to identify factors associated with CAD. Each model consists of only one of the parameters from ABPM (e.g., 24 h SBP or 24 h DBP, etc.) and the same set of potential cofounding factors (age, smoking, hypertension, TG, statins using, hemodialysis). ABPM: ambulatory blood pressure monitoring; CAD: coronary artery disease; OR: odds ratio; CI: confidence interval; SBP: systolic blood pressure; DBP: diastolic blood pressure; PP: pulse pressure (SBP—DBP). Significant differences are marked in bold.

## Data Availability

The data presented in this study are available on request from the corresponding author.

## Supplementary Materials

The following supporting information can be downloaded at: https://www.mdpi.com/article/10.3390/diagnostics13172724/s1, Table S1: Selected ABPM parameters in patients with and without CAD; Table S2: Correlations between selected ABPM parameters and CAD; Table S3: Correlations between ABPM parameters and potential predictors of CAD.
