# Supplementary material for: Ambulatory Blood Pressure Monitoring as a Useful Tool in the Cardiological Assessment of Pancreas Transplant Recipients with Type 1 Diabetes
Source: Diagnostics (Basel). 2023 Aug 22;13(17):2724. doi: 10.3390/diagnostics13172724 (PMC10487007; doi:10.3390/diagnostics13172724)
Supplement: Supplementary file 1 [file diagnostics-13-02724-s001.zip › diagnostics-2452538-supplementary.pdf]

Supplementary Table S1. Selected ABPM parameters in patients with and without CAD.

	Total (n=86)	CAD (n=26)	No CAD (n=60)	P-value
24-hour SBPL [%]	39 (18 – 71)	53.5 (31 - 91)	33 (12 – 51)	**0.002**
24-hour DBPL [%]	10.5 (2 – 30)	17.5 (5 – 36)	8.5 (2 – 29)	0.1
Isolated Systolic Hypertension [n]	20 (23%)	8 (31%)	12 (20%)	0.3
Isolated Diastolic Hypertension [n]	1 (1.1%)	0	1 (1.7%)	1.0
Systolic-Diastolic Hypertension [n]	27 (31%)	11 (42.3%)	16 (26.7%)	0.2
Dippers [n]	13 (15.1%)	6 (23.1%)	7 (11.7%)	0.2

Supplementary Table S1. Categorical variables are presented as numbers and percentages (%), and continuous variables are presented as median with interquartile range (IQR). ABPM, Ambulatory blood pressure monitoring; CAD, coronary artery disease, SBPL, systolic blood pressure load; DBPL, diastolic blood pressure load; Isolated Systolic Hypertension, participants with 24-hour systolic BP > 130mmHg and diastolic BP ≤ 80mmHg; Isolated Diastolic Hypertension, participants with 24-hour systolic BP ≤ 130mmHg and diastolic BP > 80mmHg; Systolic-Diastolic Hypertension, participants with 24-hour systolic BP > 130mmHg and diastolic BP > 80mmHg; Dippers, participants with normal SBP dipping status (a ratio of night-to-daytime SBP ≤ 0.9); Significant differences are marked in bold.

Supplementary Table S2. Correlations between selected ABPM parameters and CAD.

	Spearman's correlation coefficient	P-Value
24-hour SBPL	**0.34**	**0.001**
24-hour DBPL	0.16	0.13
Isolated Systolic Hypertension	0.12	0.28
Isolated Diastolic Hypertension	- 0.07	0.51
Systolic-Diastolic Hypertension	0.15	0.15
Dippers	0.14	0.18
24-hour SBP	**0.35**	**0.001**
24-hour DBP	0.19	0.08

Supplementary Table S2. ABPM, Ambulatory blood pressure monitoring; CAD, coronary artery disease, SBPL, systolic blood pressure load; DBPL, diastolic blood pressure load; Isolated Systolic Hypertension, participants with 24-hour systolic BP > 130mmHg and diastolic BP ≤ 80mmHg; Isolated Diastolic Hypertension, participants with 24-hour systolic BP ≤ 130mmHg and diastolic BP > 80mmHg; Systolic-Diastolic Hypertension, participants with 24-hour systolic BP > 130mmHg and diastolic BP > 80mmHg; Dippers, participants with normal SBP dipping status (a ratio of night-to-daytime SBP ≤ 0.9). Classification of the correlation strength: 0.0 ≤ |r| ≤ 0.2, no correlation; 0.2 ≤ |r| ≤ 0.4, low correlation; 0.4 ≤ |r| ≤ 0.7, moderate correlation; 0.7 ≤ |r| ≤ 0.9, high correlation; 0.9 ≤ |r| ≤ 1.0, very high correlation. Significant differences are marked in bold. Significant differences are marked in bold.

Supplementary Table S3. Correlations between ABPM parameters and potential predictors of CAD.

	**Age**	**Smoking**	**Triglycerides**	**Statins using**	**Hemodialysis**
24–hour SBP	r = 0.07	**r = 0.26**	r = 0.13	r = 0.13	**r = 0.37**
	p = 0.54	**p = 0.02**	p = 0.22	p = 0.24	**p = 0.0004**
24-hour DBP	**r = - 0.25**	**r = 0.24**	r = 0.17	r = - 0.03	**r = 0.23**
	**p = 0.02**	**p = 0.03**	p = 0.13	p = 0.76	**p = 0.03**
24-hour PP	**r = 0.3**	r = 0.18	r = 0.05	**r = - 0.23**	**r = 0.37**
	**p = 0.01**	p = 0.1	p = 0.66	**p = 0.03**	**p = 0.0004**
Daytime SBP	r = 0.06	**r = 0.28**	r = 0.13	r = 0.11	**r = 0.35**
	p = 0.61	**p = 0.01**	p = 0.23	p = 0.3	**p = 0.001**
Daytime DBP	**r = - 0.25**	r = 0.21	r = 0.13	r = - 0.06	r = 0.2
	**p = 0.02**	p = 0.05	p = 0.24	p = 0.22	p = 0.06
Daytime PP	**r = 0.29**	r = 0.18	r = 0.06	**r = 0.13**	**r = 0.38**
	**p = 0.01**	p = 0.1	p = 0.58	**p = 0.04**	**p = 0.0003**
Night-time SBP	r = 0.09	r = 0.18	r = 0.12	r = 0.15	**r = 0.43**
	p = 0.43	p = 0.11	p = 0.18	p = 0.17	**p = 0.00003**
Night-time DBP	r = - 0.15	r = 0.11	r = 0.11	r = 0.03	**r = 0.41**
	p = 0.17	p = 0.3	p = 0.22	p = 0.76	**p = 0.00009**
Night-time PP	**r = 0.24**	r = 0.19	r = 0.04	r = 0.2	**r = 0.35**
	**p = 0.03**	p = 0.09	p = 0.72	p = 0.07	**p = 0.001**

Supplementary Table S3. ABPM, Ambulatory blood pressure monitoring; SBP, systolic blood pressure; DBP, diastolic blood pressure; PP, pulse pressure (SBP - DBP). r, Spearman's correlation coefficient; p, P-Value; Classification of the correlation strength: $0.0 \leq |r| \leq 0.2$, no correlation; $0.2 \leq |r| \leq 0.4$, low correlation; $0.4 \leq |r| \leq 0.7$, moderate correlation; $0.7 \leq |r| \leq 0.9$, high correlation; $0.9 \leq |r| \leq 1.0$, very high correlation. Significant differences are marked in bold.
